# Supplementary material for: Dimensionality-dependent electronic and vibrational dynamics in low-dimensional organic-inorganic tin halides
Source: Nat Commun. 2026 Jan 15;17:758. doi: 10.1038/s41467-026-68544-8 (PMC12820188; doi:10.1038/s41467-026-68544-8)
Supplement: Supplementary file 1 — Supplementary Information [file 41467_2026_68544_MOESM1_ESM.pdf]

Supporting Information

Dimensionality-dependent electronic and vibrational dynamics in low-dimensional organic-inorganic tin halides

Yanmei He[1], Xinyi Cai[2], Rafael B. Araujo[3], Yibo Wang[1,4], Sankaran Ramesh[1], Junsheng Chen[5], Muyi Zhang[2], Tomas Edvinsson[3], Feng Gao[2,], Tönu Pullerits[1,*]*

[1] Division of Chemical Physics and NanoLund, Lund University, P.O. Box 124, 22100 Lund, Sweden

[2] Department of Physics, Chemistry, and Biology (IFM), Linköping University, SE-581 83 Linköping, Sweden

[3] Department of Materials Science and Engineering – Solid State Physics, Uppsala University, Box 534, Uppsala SE-75121, Sweden

[4] Department of Nuclear Science & Technology, Nanjing University of Aeronautics and Astronautics, Nanjing 211106, China

[5] Nano-Science Center & Department of Chemistry, University of Copenhagen, Universitetsparken 5, 2100 Copenhagen, Denmark

*Corresponding authors: tonu.pullerits@chemphys.lu.se; feng.gao@liu.se

This file contains:

Supplementary Figures 1 – 37

Supplementary Tables 1 – 7

Supplementary Note 1 – 6

Supplementary References 1 – 39

Supplementary Table 1. The peak analysis results of the 2D system

Supplementary Table 2. The fitting results from the SVD method used in the transient absorption data

Supplementary Table 3. Fitting parameters for the oscillatory signals after subtracting the population dynamics from the TA spectra of the 2D system

Supplementary Table 4. Fitting parameters for the oscillatory signals after subtracting the population dynamics from the TA spectra of the 1D system

Supplementary Table 5. Summary of experimental vibrational modes at different temperatures

Supplementary Table 6. Exciton-phonon coupling parameters are determined from the band-edge GSB signal of the 2D system

Supplementary Table 7. Assignment of the vibrational spectrum in the 1D system

Supplementary Note 1. Experimental details of thin film preparation

Supplementary Note 2. Steady-state and time-resolved spectra

Supplementary Note 3. Correlation between exciton dynamics and excitation

Supplementary Note 4. Estimation of exciton-phonon coupling strength

Supplementary Note 5. Analysis of temperature-dependent lattice dynamics

Supplementary Note 6. Theoretical calculation results

Supplementary Note 1 Experimental details of thin film preparation

The organic ligands are synthesized according to the previous report.[1,2] Octylene diammonium diodide (ODA-I_2) and SnI_2 were dissolved in anhydrous DMF at volume ratios of 2:1 or 4:1 to prepare precursor solutions with a concentration of 1 mol/mL. Quartz substrates were cleaned with detergent and subjected to ultrasonic cleaning in deionized water for 10 minutes. After drying with high-speed nitrogen flow, the substrates underwent 10 minutes of treatment in an ultraviolet-ozone cleaner. The cleaned substrates were then transferred into a nitrogen-filled glovebox for film preparation. The precursor solution was spin-coated onto the substrates using a two-step process: spinning at 5000 or 3000 rpm for 60 s while chlorobenzene was dropped as an anti-solvent at 35 s. Finally, the films were annealed at 75°C for 8 minutes within the glovebox.

Supplementary Note 2 Steady-state and time-resolved spectra

Energy gap fluctuations arise from the coupling between the electronic state and nuclear vibrations (phonons). The strength of exciton-phonon coupling (EPC) is typically quantified by the Huang-Rhys factor S, which describes the displacement between the harmonic potential energy surfaces of the ground and excited states. Since energy gap fluctuations are closely tied to exciton-phonon interaction, we present a detailed derivation of EPC strength calculation based on the displaced harmonic oscillator model.

The energy of an electronic state in the presence of nuclear motion is given by:[3]

$$E_i = E_0 + gQ \tag{1}$$

Where E_0 is the equilibrium energy gap, Q is the nuclear displacement coordinate and g is the linear exciton-phonon coupling coefficient, defined as:

$$g = m\omega^2 Q_e \tag{2}$$

where Q_e represents the displacement between the potential energy surfaces of the ground state and the excited state.

The energy gap fluctuation is defined as:

$$\delta\omega_i = E_i - \langle E_i \rangle \tag{3}$$

And its variance is given by:

$$\langle (\delta\omega_i)^2 \rangle = g^2 \langle Q^2 \rangle \tag{4}$$

For a quantum harmonic oscillator with vibrational frequency ω, the mean square displacement at finite temperature is[4,5]

$$\langle Q^2 \rangle = \frac{\hbar}{2m\omega}(2n_T + 1) \tag{5}$$

where n_T is the Bose-Einstein occupation number:[6, 7, 8]

$$n_T = \frac{1}{e^{\hbar\omega/k_BT} - 1} \tag{6}$$

The Huang-Rhys factor S factor is defined as:[9]

$$S = \frac{1}{2} m\omega \frac{{Q_e}^2}{\hbar} \tag{7}$$

Thus, the root mean square energy gap fluctuations can be expressed by:

$$\Delta(T) = \sqrt{\langle(\delta\omega_i)^2\rangle} = \hbar\omega\sqrt{S \cdot (2n_T + 1)} \tag{8}$$

At zero temperature ($T = 0$, $n_T = 0$), the energy gap fluctuation reduces to:

$$\Delta(T) = \hbar\omega\sqrt{S} \tag{9}$$

At high temperature limit ($k_BT \gg \hbar\omega$, where $n_T \approx k_BT/\hbar\omega \gg 1$), the energy fluctuation simplifies to:

$$\Delta(T) = \sqrt{2Sk_BT\hbar\omega} \tag{10}$$

These equations indicate that energy gap fluctuations directly influence the spectral linewidth and explain the temperature dependence of fluorescence spectra. By measuring the energy gap fluctuations at a given temperature, the Huang-Rhys factor can be determined, thereby quantifying the strength of EPC. We point out that often the eq. (8) is cast in the form $\Delta(T) = \hbar\omega\sqrt{S \cdot \coth(\hbar\omega/2k_BT)}$. Also, instead of the root mean square, in spectroscopy often FWHM is used. For Gaussian bands they are related as $\text{FWHM} = 2.36 \cdot \Delta$.

Temperature-dependent PL measurements reveal that the linewidth of the main emission peak broadens as the temperature increases due to the enhanced phonon-phonon scattering (Supplementary Fig. 6). We noticed that the observed deviation from the expected monotonic narrowing of the PL linewidth below ~150 K in the 2D sample likely reflects the onset of additional inhomogeneous broadening mechanisms that become relevant at low temperatures (Supplementary Fig. 7). This abnormal behavior could be induced by localized disorder, phase heterogeneity, or the scattering from the impurities at low temperature, which are well-explored in the typical lead halide perovskites, such as $MAPbBr_3$ and $MAPbI_3$.[10, 11, 12, 13, 14] While we have consistently observed this behavior across multiple measurements, a more conclusive mechanistic understanding would require further analysis, which lies beyond the scope of the present study. Above 200K, a weak emission tail appears on the red side of the spectrum. The Lorentzian peak fitting of this data identifies two peaks centered at 616 nm and 660 nm, suggesting the presence of a surface trap state (SUT). The energy level of the SUT should be slightly higher than the FE state since the PL intensity of the peak at 660 nm increases with higher temperature, implying that the aid of thermal energy (26 meV) can surmount the energy

barrier between FE and SUT. We calculate the activation energy (E_a) using a typical Arrhenius equation:[15]

$$I(T) = \frac{I_0}{1+Ae^{-E_a/k_BT}} \tag{11}$$

Where $I(T)$ and I_0 are the integrated PL intensities at temperature T and 0 K, respectively. A, E_a, and k_B represents the constant, the activation energy, and the Boltzmann constant, respectively. We obtain the activation energy of the 1D system to be ~ 412 meV, which is nearly six times higher than that of the 2D system (~ 75 meV). Here, we want to mention that the E_a is estimated from the experimental data above 175 K in the 1D system, where the thermally assisted non-radiative recombination process is involved. The fitting result shows severe deviation if the data below 175 K are included, which can be due to the observed PL emission being a composite one coming from at least two concurrent reactions showing different temperature dependence.[16]

The energy spacing (~361 meV) and absorbance ratios between the 508 nm and 502 nm absorption peaks in the 2D system agree with reported spectra in layered tin halide perovskites such as PEA_2SnI_4.[17, 18, 19] In the idealized hydrogen-like Wannier exciton model, the 2s and higher excitons are expected to have significantly weaker oscillator strength than 1s. However, strong dielectric confinement in thin layered system can enhance higher-lying excitonic transitions. Therefore, the 502 nm feature is assigned to the higher-lying excitonic resonances combined with the band edge transition. The exciton binding energy in the 2D system is estimated to be approximately 400 meV, by using the classic 2D hydrogen Rydberg series with energies $E_{Ns} = E_G - R_y/(N - 1/2)^2$, where the 1s and 2s exciton states are at 2.11 eV and 2.47 eV, respectively. This yields the R_y (Rydberg energy) of ~ 0.104 eV and the E_G of ~ 2.516 eV.[20] This result is consistent with the reported values from closely related 2D tin halide perovskites such as $(PEA)_2SnI_4$, $(OA)_2SnI_4$, $(BA)_2SnI_4$.[17, 21, 22, 23] The large binding energy reflects the strong dielectric confinement and reduced dimensionality in the 2D system. The clear excitonic features observed in the absorption and photoluminescence spectra are consistent with the presence of tightly bound excitons that are stable at room temperature. In the 1D system, which shows characteristic STE emission, the stronger exciton localization is expected due to enhanced lattice distortion and electron–phonon coupling. Such localization is typically associated with even larger exciton binding energies, as observed in other STE-emissive 1D or low-symmetry halide systems.[24, 25, 26] While our current measurements do not

allow direct quantification of the binding energy, the spectral characteristics suggest that exciton confinement and localization are significant in both systems.

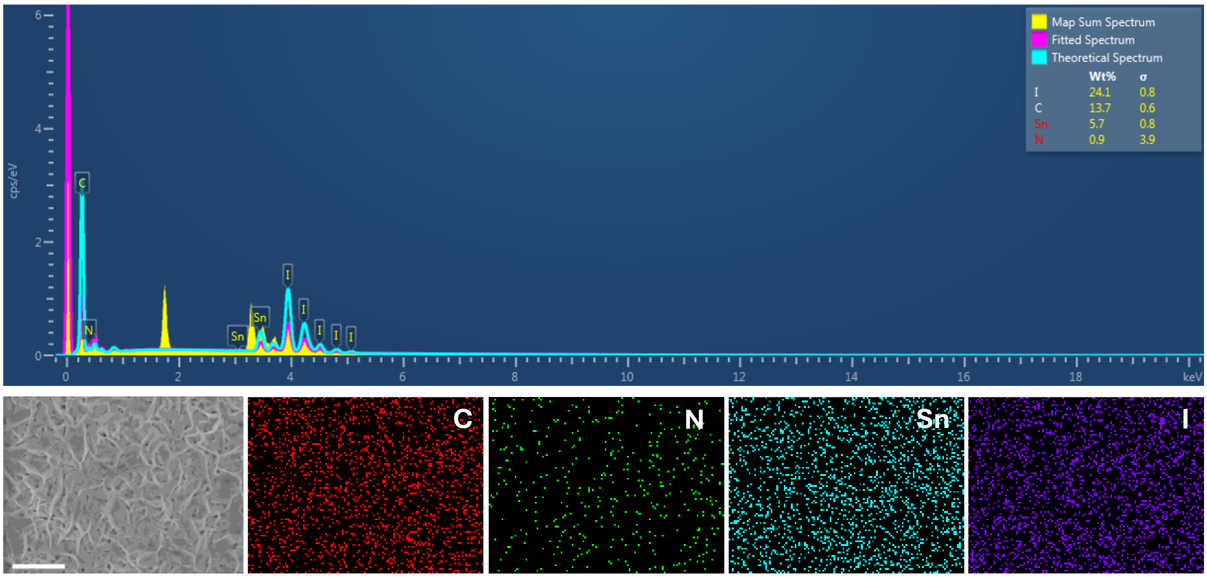

Supplementary Fig. 1 SEM images with EDS maps 2D $ODASnI_4$ thin film. These indicate the distribution of C (red), N (green), Sn (blue), and I (purple). Scale bar: 1 μm.

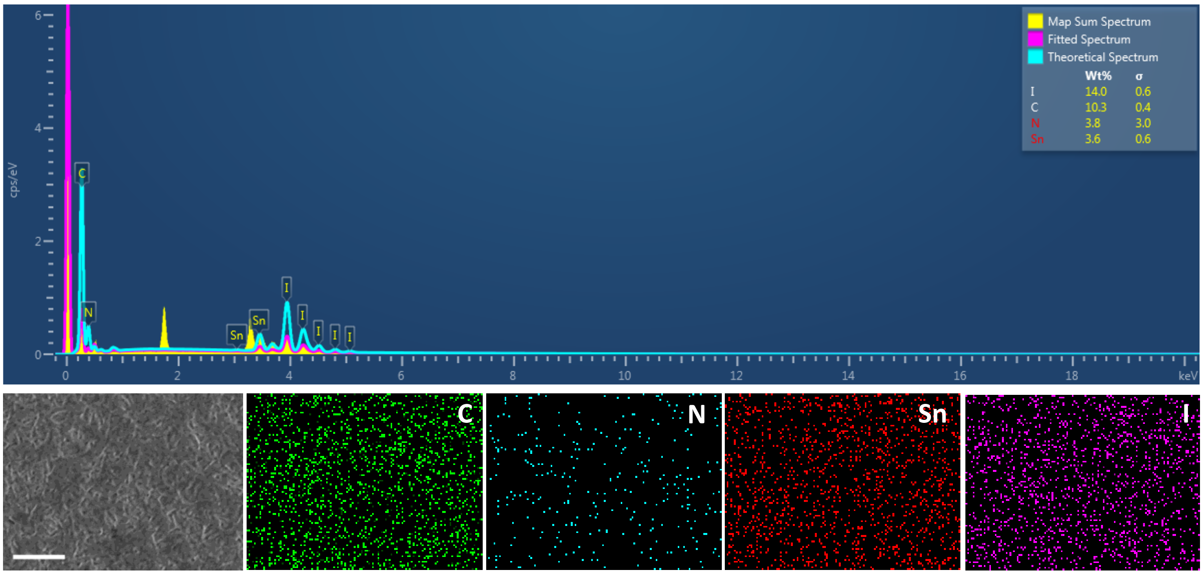

Supplementary Fig. 2 SEM images with EDS maps of 1D $ODASn_2I_6$ thin film. These indicate the distribution of C (green), N (blue), Sn (red), and I (purple). Scale bar: 1 μm.

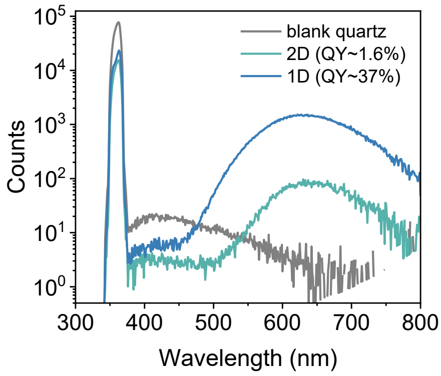

Supplementary Fig. 3 The measured PLQYs of 2D and 1D systems. The grey line represents the blank substrate.

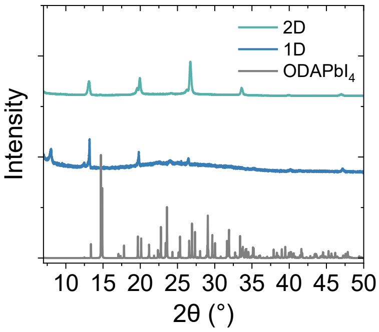

Supplementary Fig. 4 PXRD patterns of 2D and 1D systems. The standard PXRD pattern of 2D ODAPbI$_4$ is shown here for comparison.

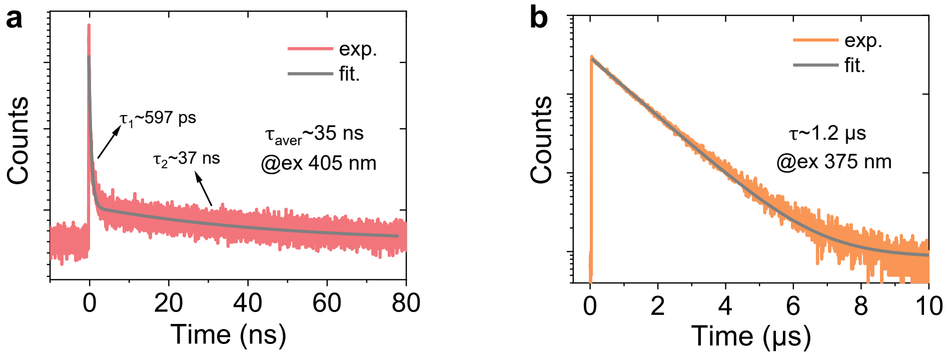

Supplementary Fig. 5 Time-resolved PL spectrum. a 2D system. **b** 1D system. The fitting results are shown inside for illustration.

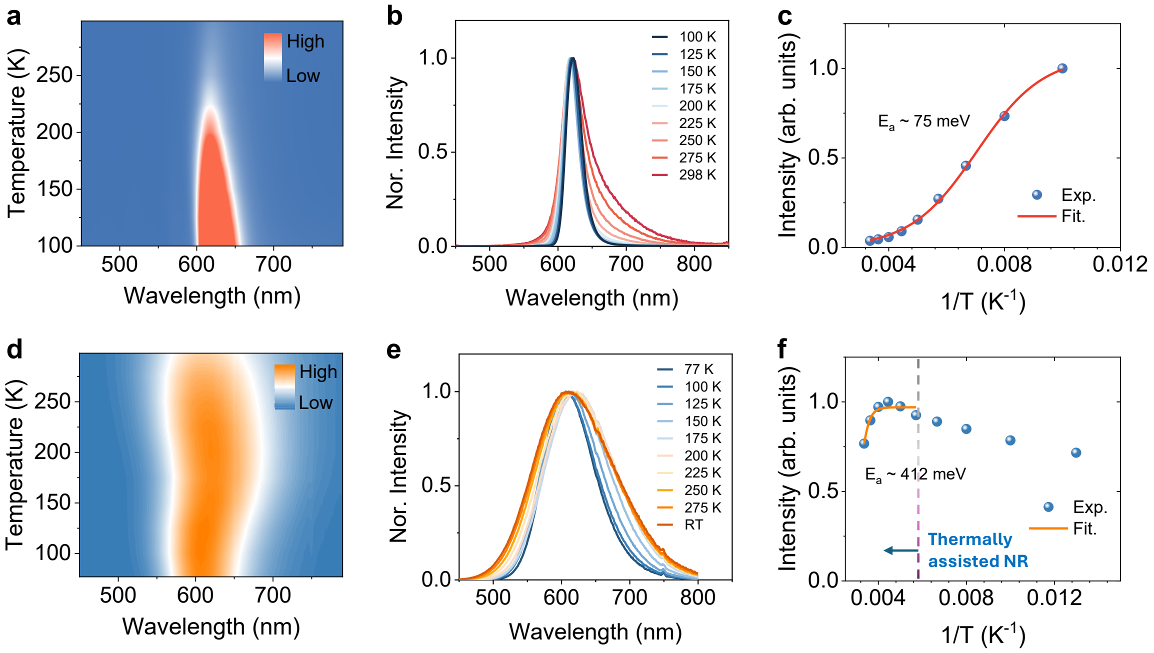

Supplementary Fig. 6 Temperature dependence analysis of emission and activation energy. Temperature-dependent PL spectra of **a-b** 2D, and **d-e** (b) 1D systems. Integrated PL intensity of the main peak as a function of temperature: **c** 2D, and **f** 1D systems. The insert is the activation energy fitted by the Arrhenius relation.

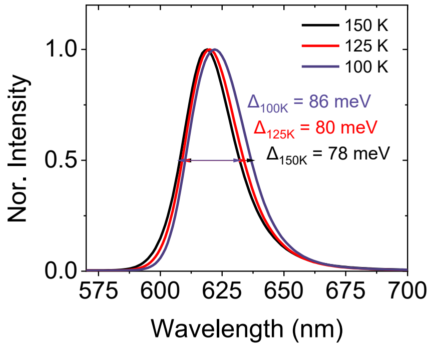

Supplementary Fig. 7 Emission linewidth analysis in the 2D system. The FWHMs of PL spectra measured at 100 K (purple), 125 K (red), and 150 K (black).

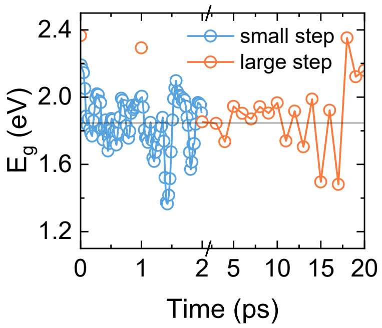

Supplementary Fig. 8 Band-gap calculation with two different MD trajectories. Blue points are the same as in Fig. 1g, the orange points are from a 20 ps MD trajectory calculated with Grimme's D3 dispersion corrections and SOC. The root mean square deviations of the calculated band gaps are 160 meV and 210 meV for the blue and orange points, respectively.

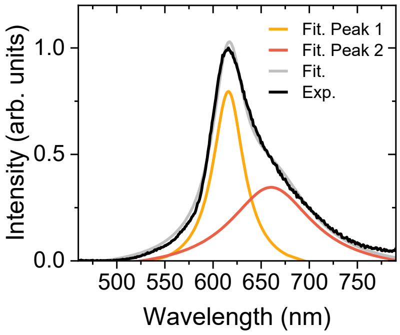

Supplementary Fig. 9 The PL spectrum of the 2D thin film with fitting results. The Lorentzian function was used for analyzing the peaks (R^2~0.996).

Supplementary Table 1. The peak analysis results are summarized for illustration.

Peak	FWHM/nm	Max Height	Center/nm	Area Integrated Proportion/%
1	39	0.85	616	48
2	105	0.40	660	52

Supplementary Note 3 Correlation of exciton dynamics and excitation

In the intensity-dependent measurements, the excitation concentration is well controlled below the threshold of the Mott transition, which is supported by the absence of blueshift around the zero-crossing in the intensity-dependent spectra (Supplementary Fig. 11).[27] In the main text, we conclude that the ultrafast component τ_1 corresponds to the hot carrier cooling, and the longer decay component reflects the combination of high-order recombination (second-order carrier recombination and Auger recombination) and trap-assisted recombination. Here, we can exclude the possibility of charge transfer between the organic ligand and inorganic moiety in the layered structure. Firstly, the absorption peaks at 502 nm and 588 nm are the intraband transition in the inorganic layers and intrinsic band-edge exciton absorption, respectively. In the TA spectra, there are two GSB signals located in similar peak positions as the steady-state absorption spectra, showing the same relaxation process. Therefore, it is not possible that the two distinct absorption peaks come from the charge transfer between the organic ligand and the inorganic moiety.[17]

Regarding the observed GSB red-shift in the TA spectra of the 2D system, we first exclude the possibility of a direct-to-indirect bandgap transition. The absorption spectrum exhibits a sharp excitonic absorption peak at the band edge, which is a characteristic signature of an allowed direct transition.[28, 29] Given the extremely small Stokes shift, we consider that radiative recombination occurs without significant phonon involvement or momentum change, further supporting a direct bandgap.[30] Here, the low PLQY does not arise from an indirect bandgap but stems from non-radiative recombination pathways (surface trap states, exciton dissociation, etc.), which are common even in direct-bandgap materials.

Secondly, BGR arises from many-body Coulomb interactions at high photoexcited carrier densities, which cause a downward shift in the conduction and valence band edges, effectively narrowing the optical bandgap (Supplementary Fig. 13b). This results in a transient red-shift of the absorption edge on ultrafast timescales, which is a well-established effect in both 3D and 2D metal halide perovskites.[31, 32] By contrast, the Burstein–Moss effect (BMS), arising from state filling and Pauli blocking, leads to a blue-shift of the absorption onset at high excitation densities (Supplementary Fig. 13a).[33, 34] The relative contributions of these two competing effects depend on excitation fluence and carrier screening. In the 2D system, we observed a small red-shift, suggesting that BGR slightly outweighs Burstein–Moss contributions.

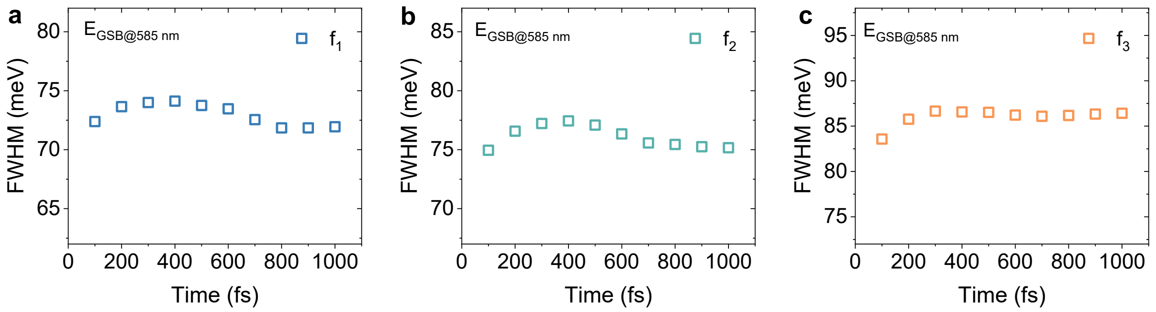

Supplementary Fig. 10 Time-evolved linewidth of GSB peak. The line width of the GSB signal (probed at 585 nm) from the lineshape analysis of the TA spectrum at the time delay of 200 fs is plotted as a function of the population time. The pump fluence ranges are **a** f_1: 3.4 × 10^{13} photon/cm^2/pulse; **b** f_2: 3.4 × 10^{14} photon/cm^2/pulse; **c** f_3: 1.7 × 10^{15} photon/cm^2/pulse.

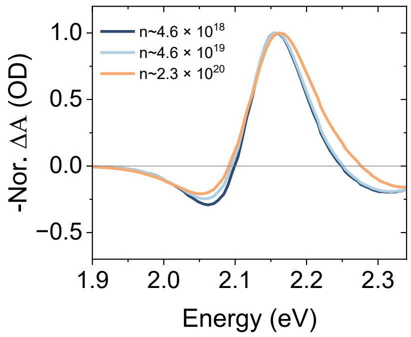

Supplementary Fig. 11 Normalized ΔA spectra probed at 585 nm (GSB signal). No blue shift of the zero-crossing is observed with increasing intensity, showing that the photocarrier concentration stays below the Mott-transition. n is the excitation density with the unit of excitation/cm^3/pulse.

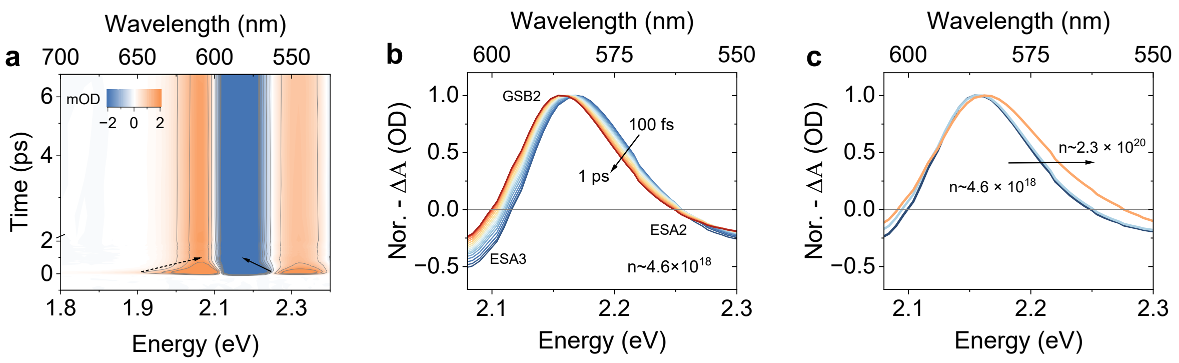

Supplementary Fig. 12 Transient absorption spectra of the 2D system. **a** Representative pseudo-color TA spectra plot of the 2D system at the first 8 ps (λ_{ex} = 400 nm). **b** Normalized TA spectra extracted from panel **a** with variable delays from 0.1 ps to 1 ps. **c** Normalized excitation-dependent TA spectra of the band-edge transition at a delay of 1 ps. n is the excitation density with the unit of excitation/cm^3/pulse.

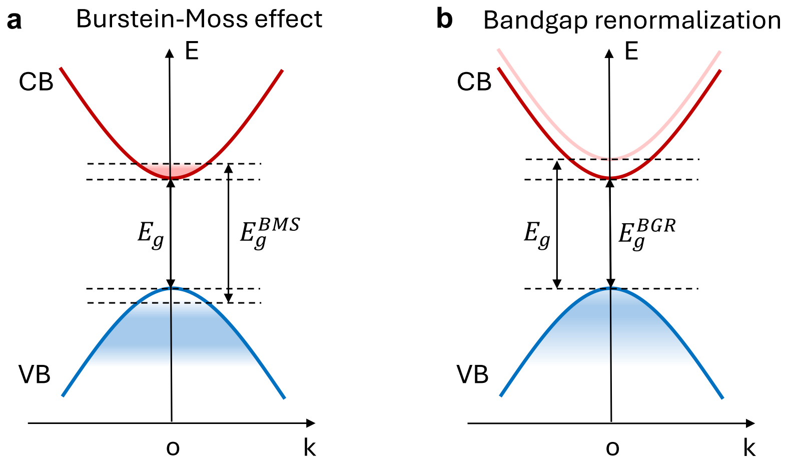

Supplementary Fig. 13 Schematic illustration of the Burstein–Moss effect and bandgap renormalization in photoexcited hybrid metal halides. **a** Following photoexcitation, state filling near the band edges (shaded region) leads to Pauli blocking and an apparent increase of the optical bandgap from E_g to E_g^{BMS} (blue-shift). **b** Many-body interactions among photogenerated carriers renormalize the band structure, reducing the bandgap from E_g to E_g^{BGR}(red-shift). CB and VB denote conduction and valence bands; E and k are energy and wavevector.

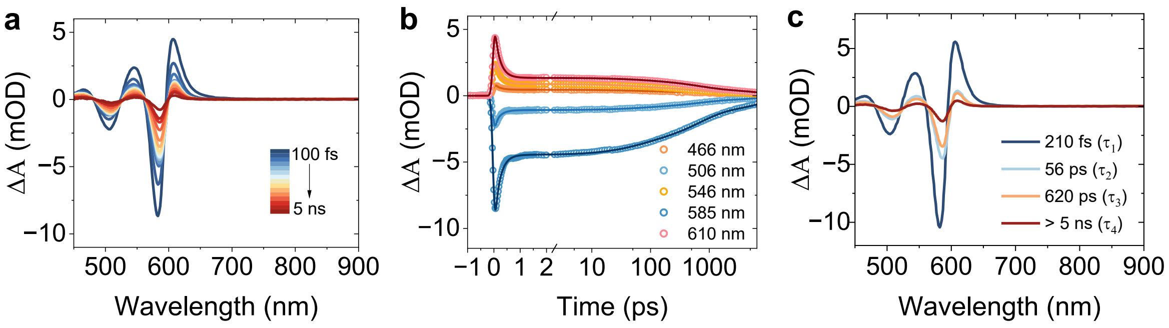

Supplementary Fig. 14 Exciton dynamics of the 2D system. **a** Spectral evolution. From green to orange: 100 fs, 300 fs, 500 fs, 800 fs, 1.5 ps, 5 ps, 10 ps, 30 ps, 50 ps, 100 ps, 200 ps, 500 ps, 1 ns, 1.5 ns, 5 ns; **b** Temporal kinetics. The solid lines represent the fitting data; **c** EAS.

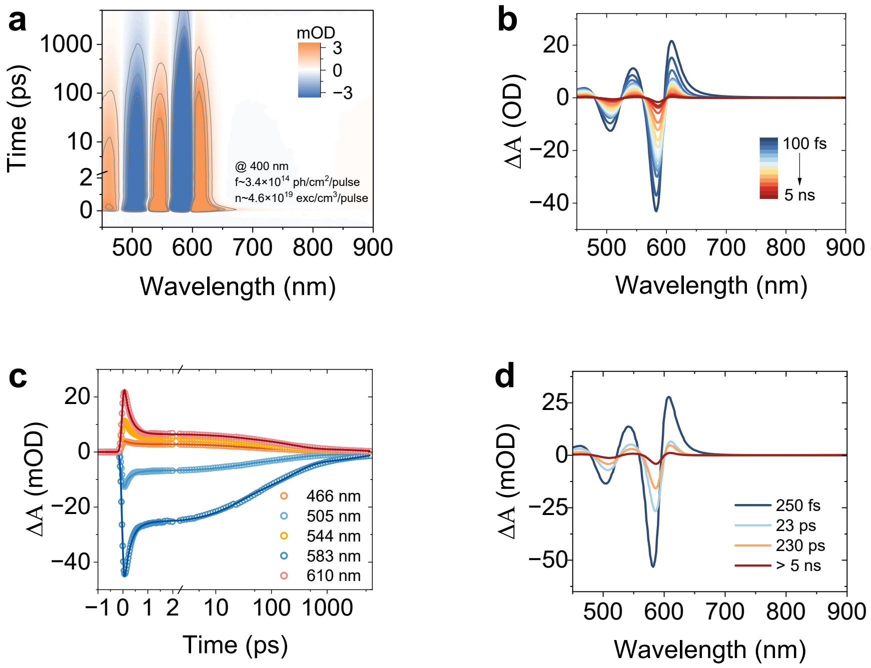

Supplementary Fig. 15 Exciton dynamics of 2D system. **a** Pseudocolor representation fs-TA spectra. **b** Spectral evolution. From green to orange: 100 fs, 300 fs, 500 fs, 800 fs, 1.5 ps, 5 ps, 10 ps, 30 ps, 50 ps, 100 ps, 200 ps, 500 ps, 1 ns, 1.5 ns, 5 ns. **c** Temporal kinetics. The solid lines represent the fitting data. **d** EAS. f: excitation fluence, photon/cm^2/pulse; n: excitation intensity, excitation/cm^3/pulse.

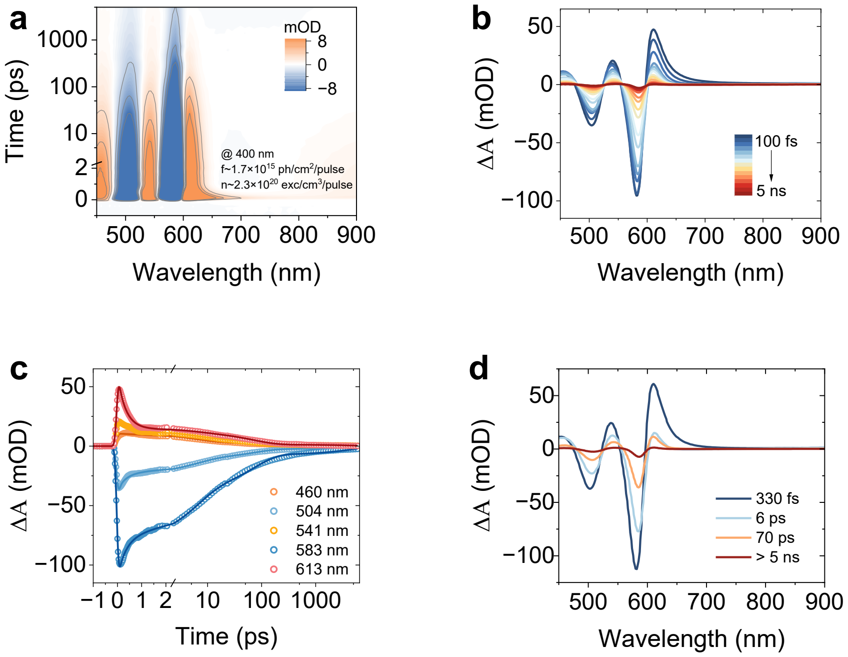

Supplementary Fig. 16 Exciton dynamics of 2D system. a Pseudocolor representation fs-TA spectra. **b** Spectral evolution. From green to orange: 100 fs, 300 fs, 500 fs, 800 fs, 1.5 ps, 5 ps, 10 ps, 30 ps, 50 ps, 100 ps, 200 ps, 500 ps, 1 ns, 1.5 ns, 5 ns. **c** Temporal kinetics. The solid lines represent the fitting data. **d** EAS. f: excitation fluence, photon/cm^2/pulse; n: excitation intensity, excitation/cm^3/pulse.

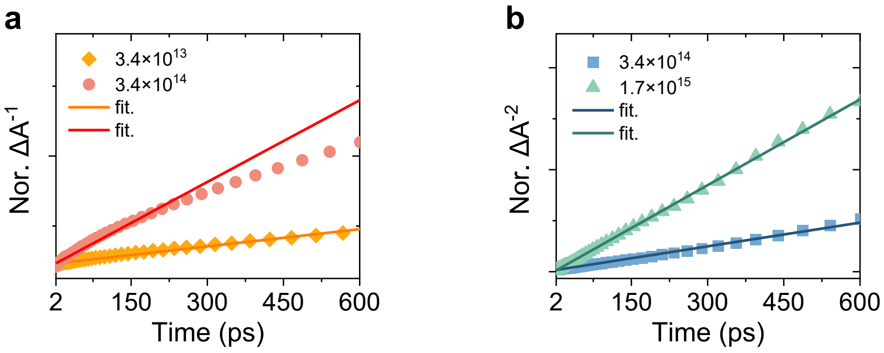

Supplementary Fig. 17 Exciton dynamics of 2D system. a normalized ΔA^{-1} and **b** normalized ΔA^{-2} vs. time at band-edge GSB kinetics with different excitation fluences (photon/cm^2/pulse). The solid lines represent the linear fitting results. Here, the Linearity in **b** indicates that the Auger recombination is dominant at the first 600 ps.

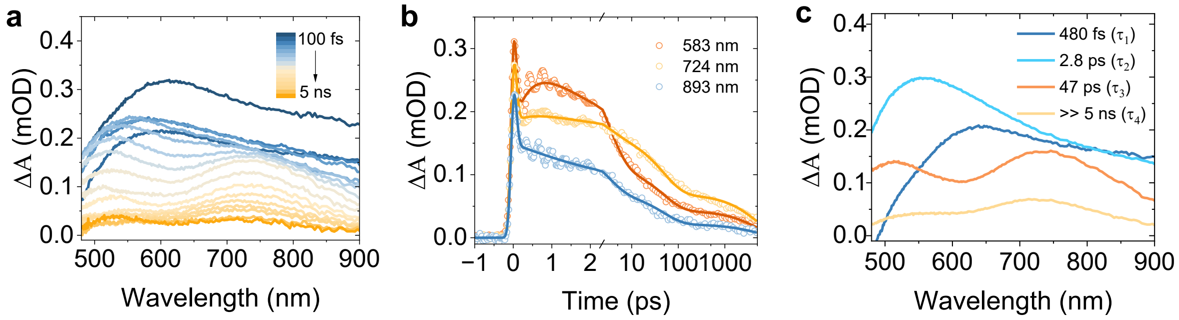

Supplementary Fig. 18 Exciton dynamics of 1D system. a Spectral evolution. From blue to yellow: 100 fs, 700 fs, 1 ps, 1.5 ps, 2.1 ps, 3 ps, 5 ps, 10 ps, 20 ps, 50 ps, 100 ps, 200 ps, 500 ps, 1 ns, 3 ns and 5 ns. **b** Temporal kinetics. The solid lines represent the fitting data. **c** EAS.

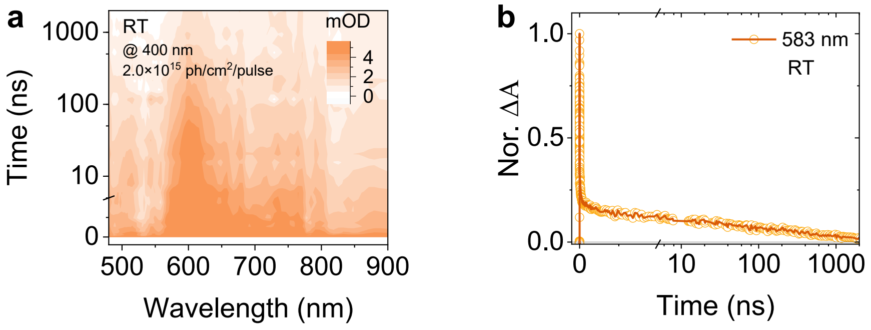

Supplementary Fig. 19 Exciton dynamics of 1D system. a TA spectrum measured at the time window ranging from 0 ns to 2000 ns. **b** The time trace is probed at 583 nm.

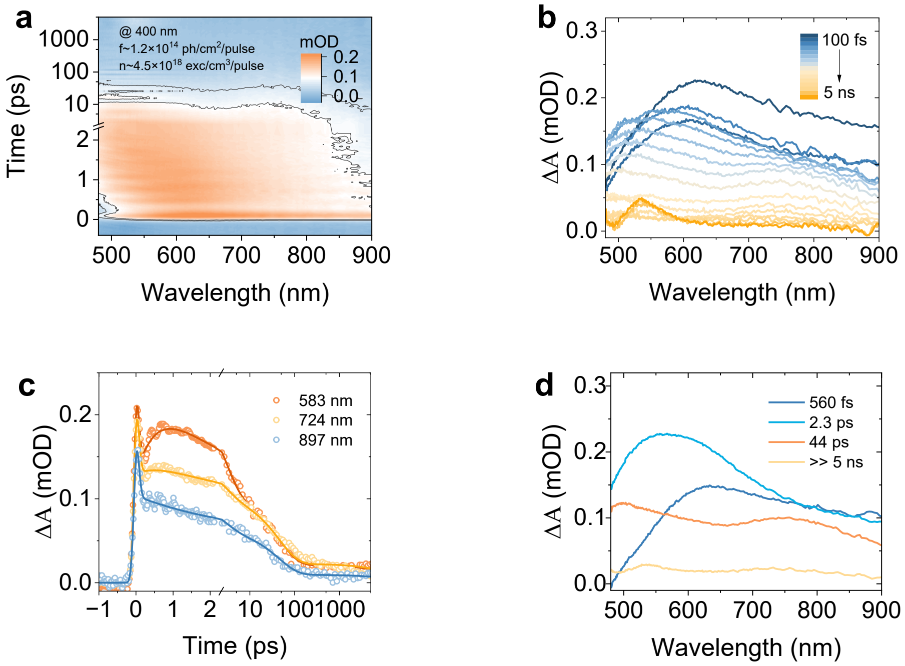

Supplementary Fig. 20 Exciton dynamics of 1D system. **a** Pseudocolor representation fs-TA spectra. **b** Spectral evolution. From blue to yellow: 100 fs, 700 fs, 1 ps, 1.5 ps, 2.1 ps, 3 ps, 5 ps, 10 ps, 20 ps, 50 ps, 100 ps, 200 ps, 500 ps, 1 ns, 3 ns and 5 ns. **c** Temporal kinetics. The solid lines represent the fitting data. **d** EAS. f: excitation fluence, photon/cm^2/pulse; n: excitation intensity, excitation/cm^3/pulse.

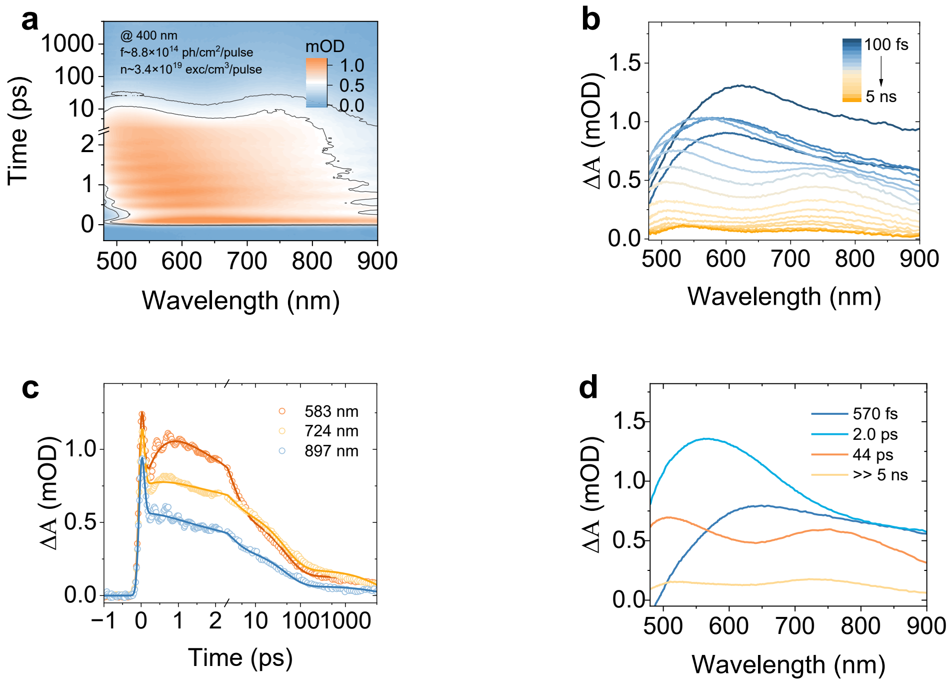

Supplementary Fig. 21 Exciton dynamics of 1D system. a Pseudocolor representation fs-TA spectra. **b** Spectral evolution. From blue to yellow: 100 fs, 700 fs, 1 ps, 1.5 ps, 2.1 ps, 3 ps, 5 ps, 10 ps, 20 ps, 50 ps, 100 ps, 200 ps, 500 ps, 1 ns, 3 ns and 5 ns. **c** Temporal kinetics. The solid lines represent the fitting data. **d** EAS. f: excitation fluence, photon/cm^2/pulse; n: excitation intensity, excitation/cm^3/pulse.

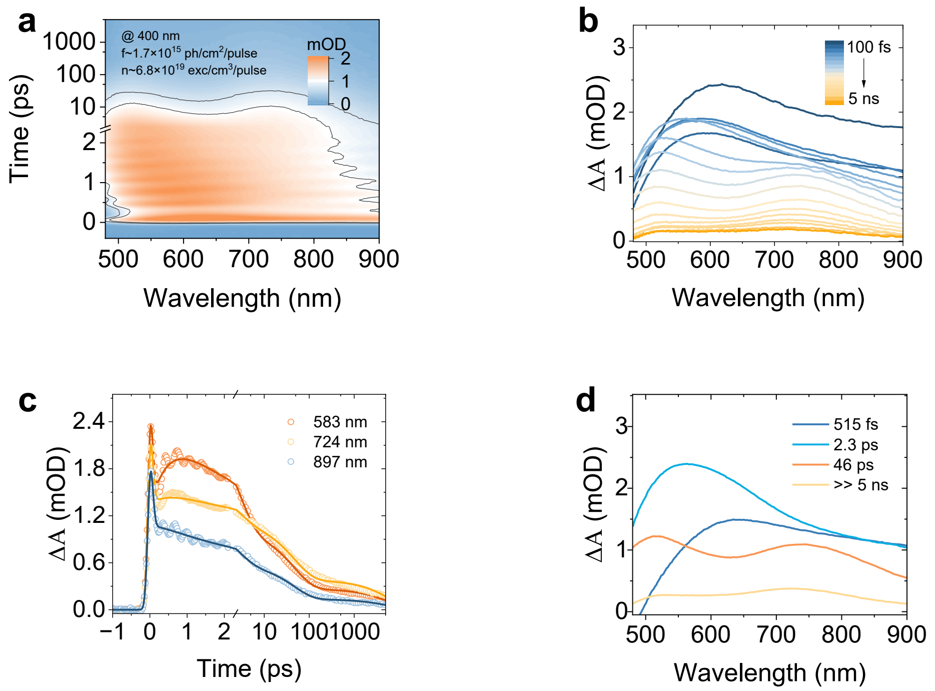

Supplementary Fig. 22 Exciton dynamics of 1D system. **a** Pseudocolor representation fs-TA spectra. **b** Spectral evolution. From blue to yellow: 100 fs, 700 fs, 1 ps, 1.5 ps, 2.1 ps, 3 ps, 5 ps, 10 ps, 20 ps, 50 ps, 100 ps, 200 ps, 500 ps, 1 ns, 3 ns and 5 ns. **c** Temporal kinetics. The solid lines represent the fitting data. **d** EAS. f: excitation fluence, photon/cm^2/pulse; n: excitation intensity, excitation/cm^3/pulse.

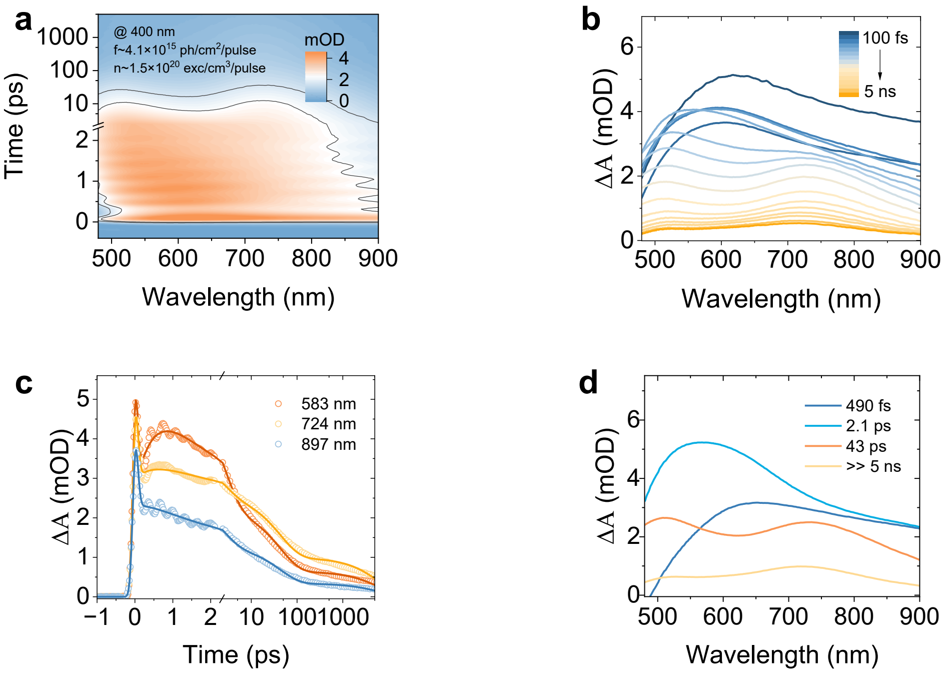

Supplementary Fig. 23 Exciton dynamics of 1D system. a Pseudocolor representation fs-TA spectra. **b** Spectral evolution. From blue to yellow: 100 fs, 700 fs, 1 ps, 1.5 ps, 2.1 ps, 3 ps, 5 ps, 10 ps, 20 ps, 50 ps, 100 ps, 200 ps, 500 ps, 1 ns, 3 ns and 5 ns. **c** Temporal kinetics. The solid lines represent the fitting data. **d** EAS. f: excitation fluence, photon/cm^2/pulse; n: excitation intensity, excitation/cm^3/pulse.

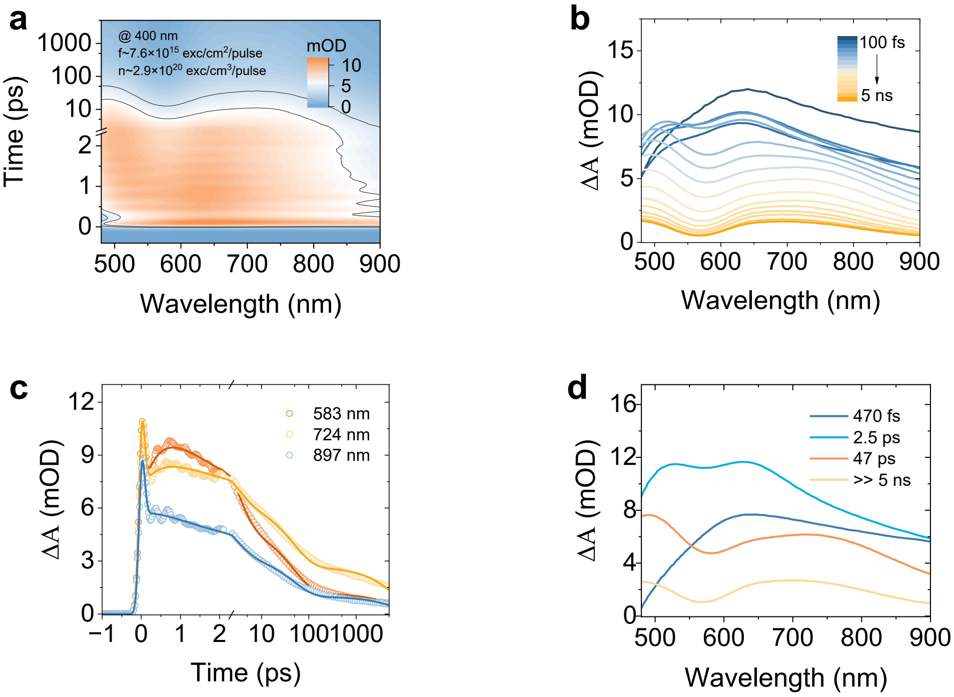

Supplementary Fig. 24 Exciton dynamics of 1D system. a Pseudocolor representation fs-TA spectra. **b** Spectral evolution. From blue to yellow: 100 fs, 700 fs, 1 ps, 1.5 ps, 2.1 ps, 3 ps, 5 ps, 10 ps, 20 ps, 50 ps, 100 ps, 200 ps, 500 ps, 1 ns, 3 ns and 5 ns. **c** Temporal kinetics. The solid lines represent the fitting data. **d** EAS. f: excitation fluence, photon/cm^2/pulse; n: excitation intensity, excitation/cm^3/pulse.

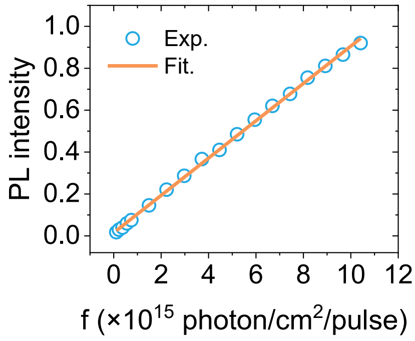

Supplementary Fig. 25 The intensity dependence of the emission. The integrated PL intensity is plotted as a function of excitation fluences in the 1D system and its linear fitting with R^2 of 0.998.

Supplementary Table 2. The fitting results from the SVD method are summarized for comparison.

Materials	Excitation fluence/f photon/cm^2/pulse	Exciton density/n excitation/cm^3/pulse	τ_1/fs	τ_2/ps	τ_3/ps	τ_4/ns
2D system	3.4×10^{13}	4.6×10^{18}	210	56	620	> 5
	3.4×10^{14}	4.6×10^{19}	250	23	230	
	1.7×10^{15}	2.3×10^{20}	330	6.0	70	
1D system	1.2×10^{14}	4.5×10^{18}	560	2.3	44	$\gg 5$
	4.1×10^{14}	1.6×10^{19}	480	2.8	47	
	8.8×10^{14}	3.4×10^{19}	570	2.0	44	
	1.8×10^{15}	6.8×10^{19}	515	2.3	46	
	4.1×10^{15}	1.5×10^{20}	490	2.1	43	
	7.6×10^{15}	2.9×10^{20}	470	2.5	47	

Supplementary Note 4 Estimation of exciton-phonon coupling strength

The pump-probe experiments use the ultrashort laser pulse to enable the observation of the periodic oscillation signals, and they are much clearer in the oscillatory signal after removing the exponential decay components. The pump pulse causes coherent oscillation of vibrational modes, leading to the collective motions of the lattice at both the ground state and the excited state. This generation mechanism is described by a damped harmonic oscillator affected by an externally applied force *F*:[35, 36]

$$\frac{d^2x}{dt^2} + 2\xi\frac{dx}{dt} + \omega_0^2 x = \frac{F}{m} \tag{12}$$

Where x, t, ξ, ω_0 and m are coherent phonon amplitude, time, damping constant, phonon frequency corresponding to a wave vector near the center of the Brillouin zone, and reduced mass, respectively. The driving force *F* is then given by:[36]

$$F = \frac{2}{\omega_0}\sum\int_{-\infty}^{\infty} d\omega \frac{E_0(\omega)E_0^*(\omega\pm\omega_0)}{\eta(\omega)\eta(\omega\pm\omega_0)}\pi^R(\omega,\omega\pm\omega_0) \times e^{-0.5[\alpha(\omega)+\alpha(\omega\pm\omega_0)]x} \tag{13}$$

Where E_0 and α are the electric field amplitude of the pump pulse and the absorption coefficient, respectively. The π^R at frequency ω is expressed by the following equation when ξ is close to 0 and $\left|\frac{\omega_0}{\omega}\right|$ is much smaller than 1:[36]

$$\pi^R(\omega+\omega_0,\omega) \approx \frac{\Xi}{4\pi\hbar}\left[\frac{d\varepsilon_{\mathrm{Re}}}{d\omega} + 2i\frac{\varepsilon_{\mathrm{Im}}}{\omega_0}\right] \tag{14}$$

Where $\varepsilon_{\mathrm{Re}}$ and $\varepsilon_{\mathrm{Im}}$ are real and imaginary parts of the dielectric constant. Ξ is the electron-phonon interaction constant via the deformation potential, which is given by $\Xi = \frac{\partial E}{\partial Q}$ (Q is nuclear coordinate). A larger electron-phonon interaction constant indicates a larger change in electronic energy with nuclear displacement. The factor η in eq. 13 has a strong relationship with the refractive index of materials:

$$\eta = n + ik + 1 = (\varepsilon_{\mathrm{Re}} + i\varepsilon_{\mathrm{Im}})^{1/2} + 1 \tag{15}$$

Where n and k are the real and imaginary parts of the refractive index, respectively.

From eq. 12-14, the coherent phonon amplitude is then given by:[37, 38]

$$x(t) \propto \frac{\varepsilon_{\mathrm{Im}}\Xi}{{\omega_a}^2} I_0 cos(\omega_a t + \varphi)e^{-\xi t} \tag{16}$$

Where the $\omega_a = \sqrt{\omega_0{}^2 - \xi^2}$, $\varphi = arctan\left(\frac{d\varepsilon_{\text{Re}}}{d\omega}\Big/\frac{2\varepsilon_{\text{Im}}}{\Omega_1}\right)$, and the pump intensity I_0 is equal to $\int_{-\infty}^{\infty}|E(t)|^2\, dt$. As a result, the coherent phonon amplitude should be linearly proportional to the pump intensity and the exciton-phonon interaction strength Ξ. Since the detection of coherent phonons is visualized by probing the absorbance of changes in the pump-probe technique, the residual amplitude of the TA signal (A_{osc}) can be expressed by the following equation:

$$\Delta A_{\text{osc}} \approx \frac{\omega_p L}{c n \ln 10}\frac{d\varepsilon_{\text{Im}}}{dE}\Xi x(t) \qquad (17)$$

Where the absorbance is calculated as $A = \frac{2\omega_p k L}{c} ln 10$, where c, ω_p and L are the speed of light, frequency of excitation, and thickness of the material, respectively. From eq. 16 and 17, we can obtain

$$\Delta A_{\text{osc}} \propto \frac{d\varepsilon_{\text{Im}}}{dE}\frac{\Xi^2}{\omega_a{}^2} I_0 \qquad (18)$$

Based on these results, the ΔA_{osc} also shows a linear dependence on the derivative of the imaginary part of the dielectric constant.

Using the displaced harmonic oscillator model (see Supplementary Fig. 26), we can show that in our case the exciton-phonon interaction strength is related to the lattice reorganization energy and the dimensionless Huang-Rhys parameter, which is used to quantify the exciton-phonon coupling. In the displaced oscillator model, the nuclear wavepacket oscillates in a harmonic potential with frequency ω_0. The lattice reorganization energy λ is expressed by[39]

$$\lambda = \frac{\Xi^2}{2m\omega_0{}^2} \qquad (19)$$

where m is the reduced mass of the phonon mode. Given eq. 18, the λ should have the linear dependence of $\frac{\Delta A_{\text{osc}}}{\left(\frac{d\varepsilon_{\text{Im}}}{dE}\right)}$. Since the derivative $\frac{d\varepsilon_{\text{Im}}}{dE}$ should be proportional to the derivative of the steady-state absorption spectrum $\frac{d\text{OD}}{dE}$, it is clear from eq. 19 that ΔA_{osc} in the TA signal should also be proportional to the reorganization energy. After normalizing the sample absorbance (OD) to the GSB intensity in the TA signal, the ΔA_{osc} can be given as $\Delta A_{\text{osc}} = \lambda \cdot \frac{d\text{OD}}{dE}$. On the other hand, the reorganization energy λ can be expressed by $S\hbar\omega_0$, which is related to the displacement ΔQ of the potential energy surfaces via the Huang-Rhys factor $S = \frac{1}{2}\Delta Q^2$.

Now, we extract the ΔA_{osc} by removing the exponential decay component from the GSB signal at 595 nm in the 2D system. The $\frac{d\mathrm{OD}}{dE}$ is the derivative of the steady-state absorption spectrum after the consistent normalization of OD based on the amplitude of the GSB signal at zero-time delay. As a result, we obtain the parameters related to the lattice vibrations, including the S, λ and ΔQ, see Supplementary Table 6. Here, we noted that the S factor of 4 cm^{-1} is overestimated. We assume this method might not be well-suited for the very low-frequency region.

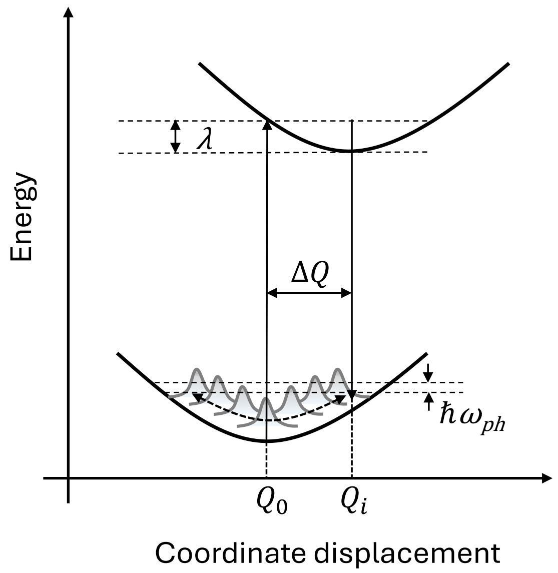

Supplementary Fig. 26 Displaced harmonic oscillator model for exciton-phonon coupling. Schematic adiabatic potential energy surfaces plotted against nuclear coordinate displacement for the ground and excited electronic states. Photoexcitation shifts the equilibrium position from Q_0 to Q_i, giving a configurational displacement ΔQ. The corresponding reorganization energy λ quantifies lattice relaxation driven by electron–phonon coupling. The grey wavepacket represents vibrational (phonon) motion along the coordinate, and $\hbar\omega_{\mathrm{ph}}$ denotes the phonon energy spacing between vibrational levels.

Supplementary Note 5 Analysis of temperature-dependent phonon coherence

As the temperature increases, the peaks in the 1D system shift to higher wavenumbers and exhibit broader linewidths due to enhanced phonon-phonon scattering (Supplementary Fig. 32). At 77 K, the M1 mode is absent, and the amplitude of vibrational modes below 70 cm^{-1} is significantly reduced due to the suppressed phonon-phonon interaction. The dephasing time of coherent phonons at 77 K is slightly different from that at room temperature. It indicates that coherent phonon dynamics are less dependent on temperatures, with the M5 mode playing a dominant role in coupling to the lattice vibration. In the 2D system, we identify three vibrational modes at 3 cm^{-1}, 24 cm^{-1}, and 47 cm^{-1} probed at 550 nm (Fig. 3i). The oscillatory signal at 595 nm involves five coherent phonon modes: 3 cm^{-1}, 14 cm^{-1}, 22 cm^{-1}, 45 cm^{-1}, and 86 cm^{-1} (Supplementary Table 3). The absence of 14 cm^{-1} and 86 cm^{-1} modes in the oscillatory signal at 550 nm suggests that phonon modes are influenced by the electronic transition.

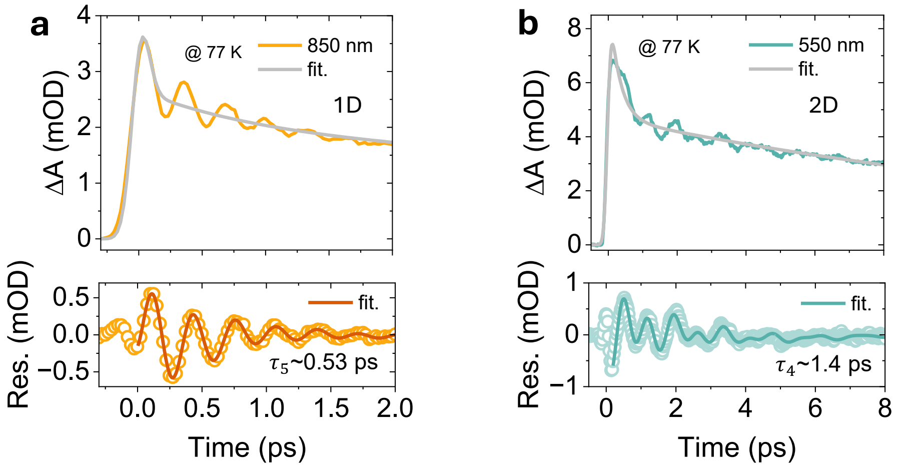

Supplementary Fig. 27 Low-temperature coherent vibrational dynamics. The temporal and oscillatory signals are extracted from the corresponding differential TA spectra of **a** 1D and **b** 2D systems. The damping time of each fitted oscillatory component is shown for comparison.

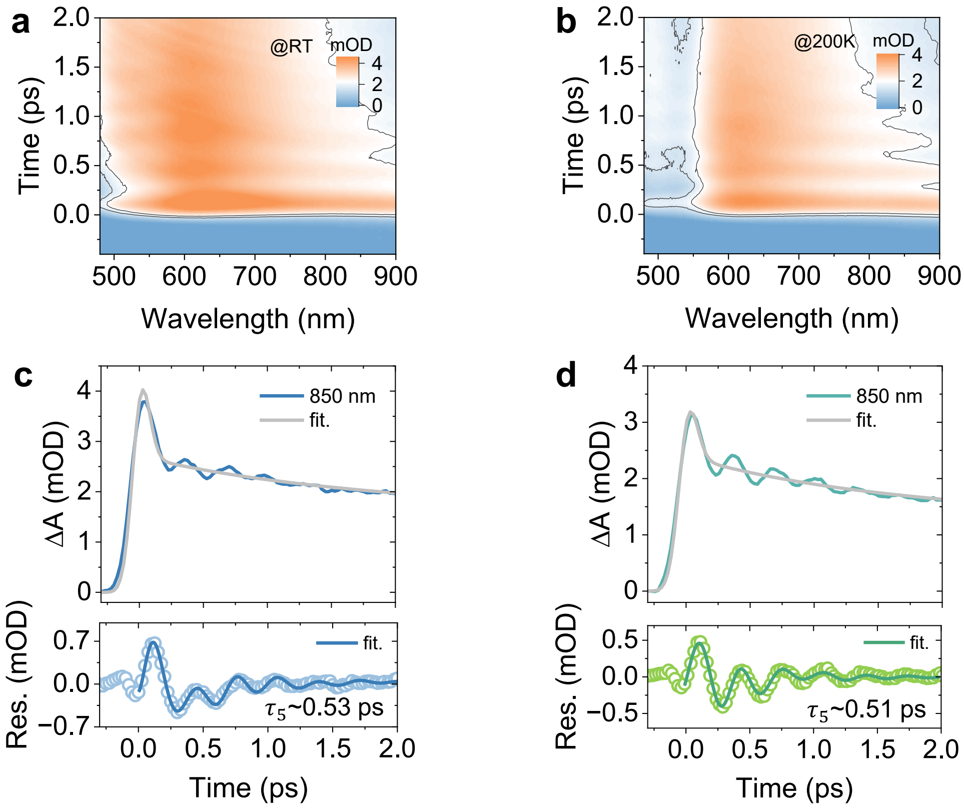

Supplementary Fig. 28 Vibrational dynamics of 1D system. Pseudocolor representation of fs-TA spectra measured at **a** RT and **b** 200 K. The temporal kinetics and oscillatory signals are extracted from the differential TA spectra measured at **c** RT and **d** 200 K.

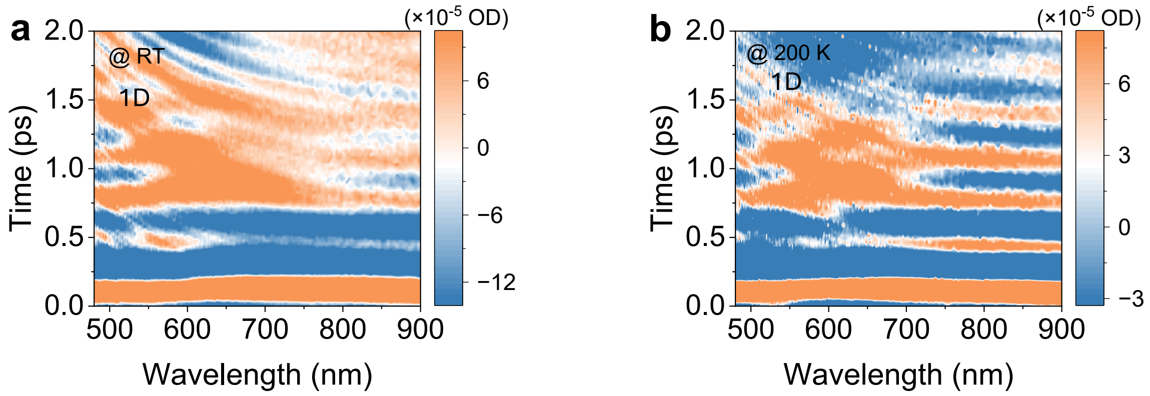

Supplementary Fig. 29 Vibrational dynamics of 1D system. Temperature-dependent coherent vibrational dynamics, which is obtained by subtracting the population dynamics: **a** at RT; **b** at 200K

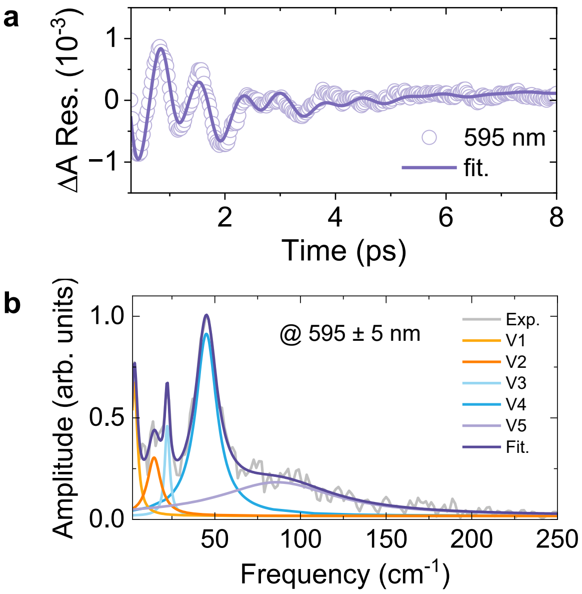

Supplementary Fig. 30 Vibrational dynamics of 2D system. **a** The oscillatory signals at 595 nm are extracted from the differential TA spectra measured at 77 K. The fitting results using the damped cosine function are shown for comparison. **b** Fourier transformed spectrum (probed at 595 ± 5 nm) extracted from Fig. 3d. The Lorentzian fitting results are shown for comparison.

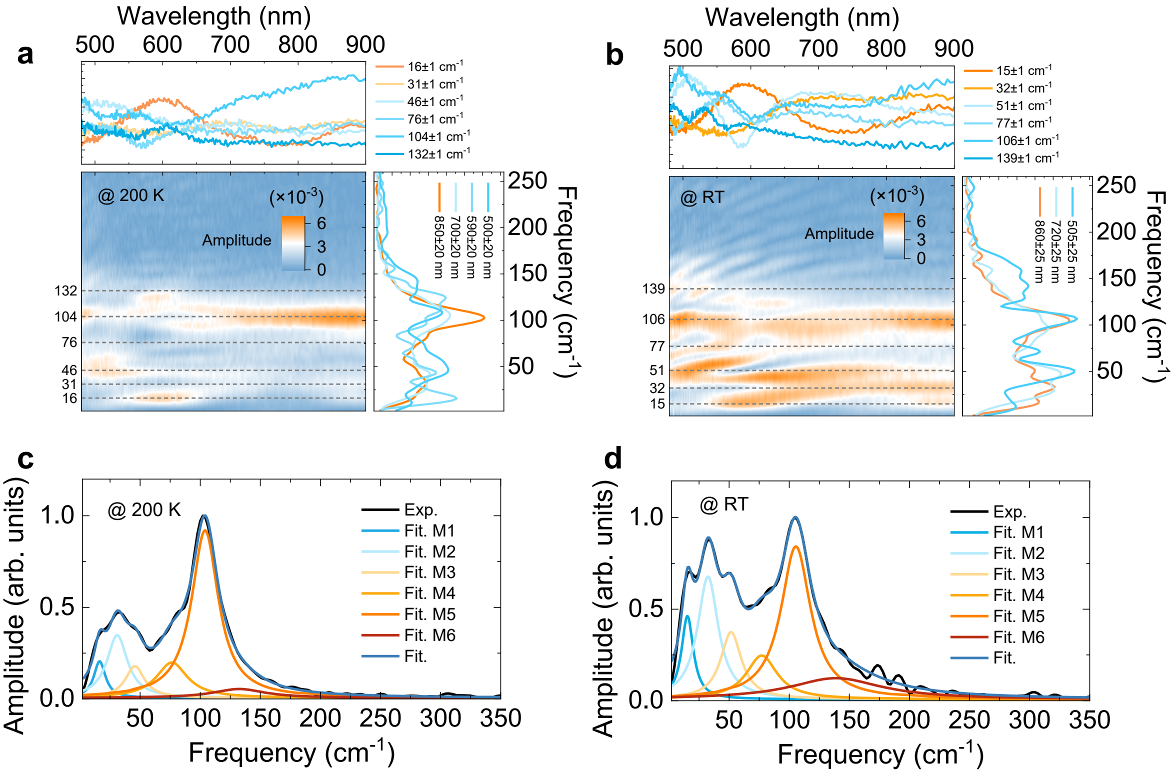

Supplementary Fig. 31 Vibrational dynamics of 1D system. Probe wavelength resolved vibrational information directly obtained by FFT of the differential TA spectrum measured at **a** 200 K and **b** RT. Vibrational spectra at **c** 200 K and **d** RT with the Lorentzian fitting results.

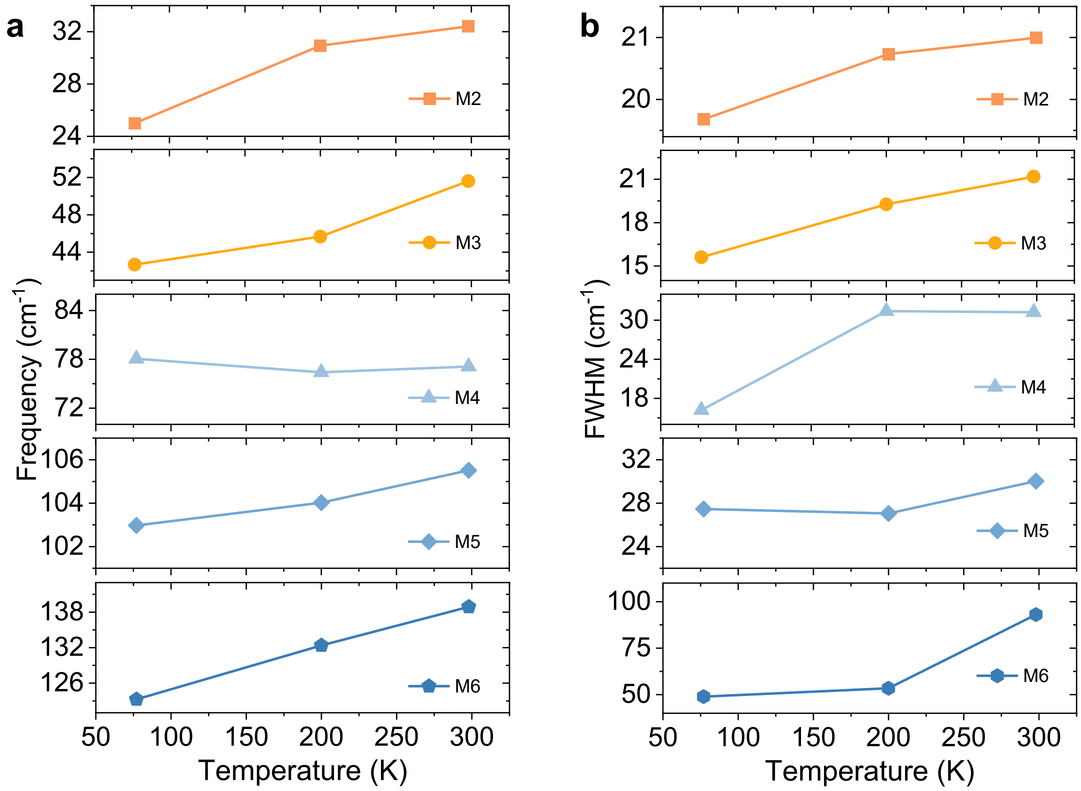

Supplementary Fig. 32 Vibrational frequency analysis of 1D system. Temperature dependence of **a** vibrational mode frequency and **b** its corresponding FWHM.

Supplementary Table 3. Fitting parameters for the oscillatory signals after subtracting the population dynamics from the TA spectra of the 2D system.

77 K					
at 595 nm (GSB)					
Components	1	2	3	4	5
A_i/mOD	0.47	0.23	-0.39	1.3	0.11
τ_i/ps	3.4	1.9	2.2	1.3	1.0
v_i/cm^{-1}	3	14	22	45	86
φ_i/rad	0.38π	1.5π	1.5π	1.8π	1.2π
at 550 nm (ESA)					
Components	1	2	3	4	5
A_i/mOD	0.15	-	0.24	0.67	-
τ_i/ps	4.2	-	3.5	1.4	-
v_i/cm^{-1}	4	-	25	46	-
φ_i/rad	1.8π	-	0.90π	0.64π	-

Supplementary Table 4. Fitting parameters for the oscillatory signals at 850 nm after subtracting the population dynamics from the TA spectra of the 1D system.

77 K						
Components	1	2	3	4	5	6
A_i/mOD	-	0.20	0.031	-0.11	0.81	0.27
τ_i/ps	-	0.55	1.5	0.37	0.53	0.10
v_i/cm^{-1}	-	24	46	72	104	123
φ_i/rad	-	1.2π	1.9π	1.2π	1.3π	0.35π
200 K						
Components	1	2	3	4	5	6
A_i/mOD	0.23	0.24	0.11	-0.09	0.46	0.10
τ_i/ps	0.18	0.39	0.45	0.32	0.51	0.36
v_i/cm^{-1}	16	31	46	76	102	132
φ_i/rad	0.67π	0.13π	1.5π	0.70π	1.4π	0.51π
RT						
Components	1	2	3	4	5	6
A_i/mOD	-0.66	0.69	-0.11	0.46	0.65	-0.20
τ_i/ps	0.43	0.39	0.52	0.23	0.53	0.25
v_i/cm^{-1}	15	32	52	76	106	138
φ_i/rad	1.2π	0.23π	1.5π	0.83π	1.1π	0.14π

Supplementary Table 5. Summary of experimental vibrational modes at different temperatures.

1D system							
Temperatures (K)	Mode Frequency/FWHM (cm^{-1})						
	Probe wavelength	M_1	M_2	M_3	M_4[1]	M_5	M_6
77	850 ± 25 nm	-/-	24/20	43/16	78/16	103/27	123/49
200		16/12	31/21	46/19	76/31	104/27	132/53
RT		15/12	32/21	52/21	77/31	106/30	139/93
2D system							
Temperatures (K)	Mode Frequency/FWHM (cm^{-1})						
	Probe wavelength	V_1	V_2	V_3	V_4	V_5	-
77	595 ± 5 nm	3/4	14/9	22/3	45/15	86/82	-
	550 ± 25 nm	3/4	-	24/6	47/11	-	-

Supplementary Table 6. Exciton-phonon coupling parameters are determined from the band-edge GSB signal of the 2D system.

Probe wavelength	ω_{ph} (cm^{-1})	λ (meV)	S	ΔQ
595 nm (at 77 K)	3	2.6	7.0	3.7
	14	1.3	0.74	1.2
	22	2.2	0.80	1.3
	45	7.4	1.3	1.6
	86	0.61	0.06	0.34

Supplementary Note 6 Theoretical calculation results

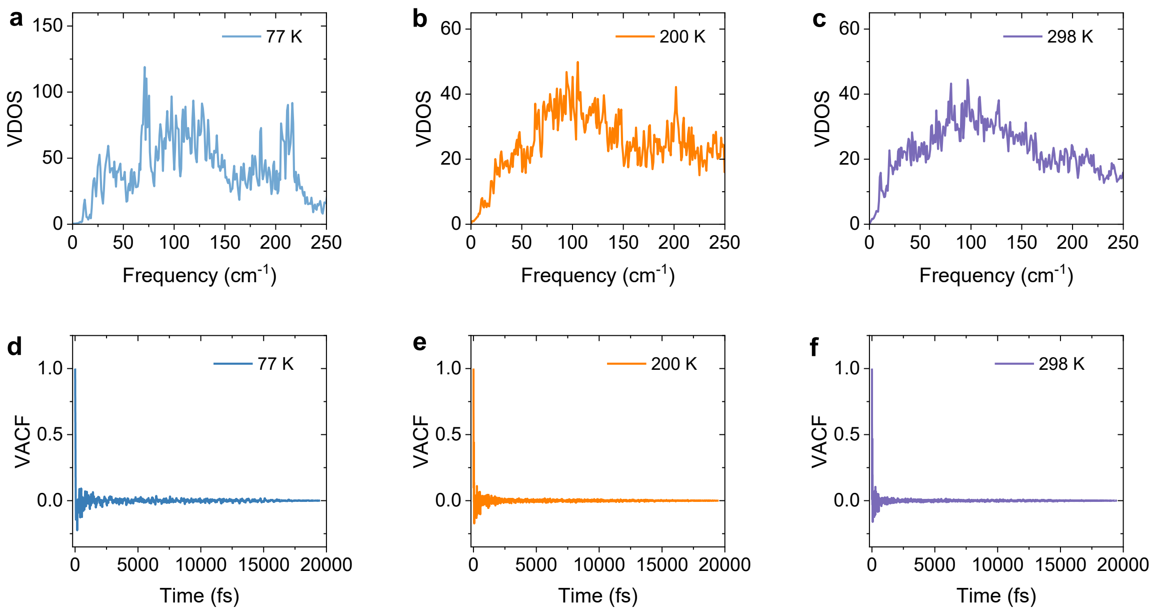

Supplementary Fig. 33 The structural vibrations and mode frequencies visualized by the AIMD simulations. The VDOS and VACF of the 1D system. 77K: **a** and **d**; 200K: **b** and **e**; 298K: **c** and **f**.

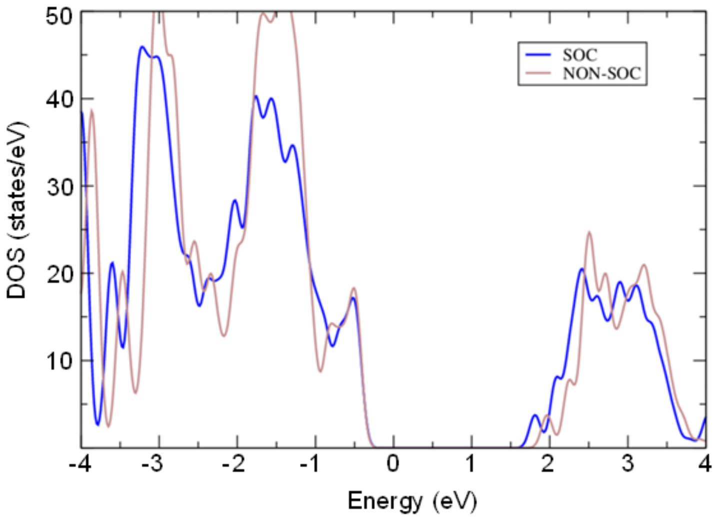

Supplementary Fig. 34 Total DOS using PBE functional, considering SOC and without SOC. The results showed only minor differences in the electronic structure (the maximum deviation in the band gap is approximately 0.15 eV); thus, for consistency, the band structure calculations were performed using the non-spin-polarized approach.

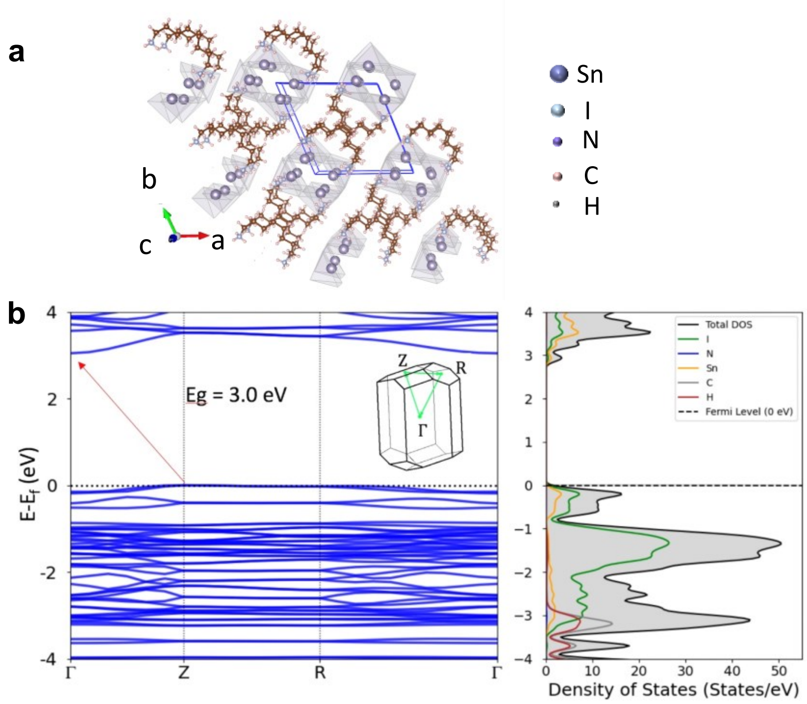

Supplementary Fig. 35 Theoretical calculation. a Crystal structure of the 1D system: primitive unit cell. **b** Band structure and PDOS of 1D system with HSE06 level of theory.

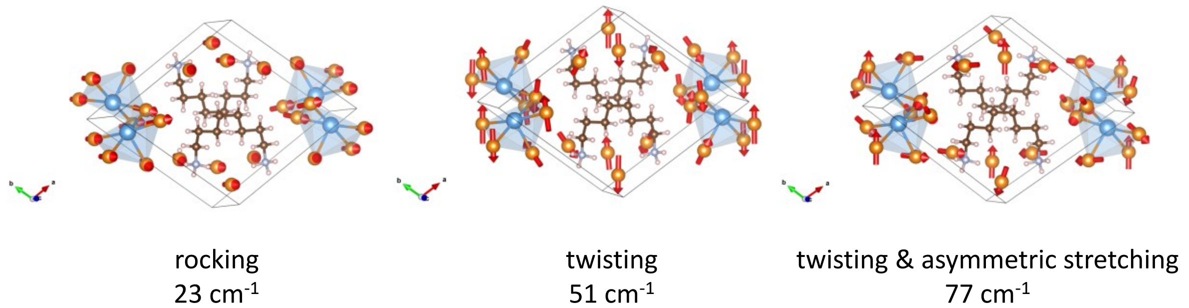

Supplementary Fig. 36 Simulated mode frequencies in the 1D system. The low-frequency vibrational modes and their associated atomic motions.

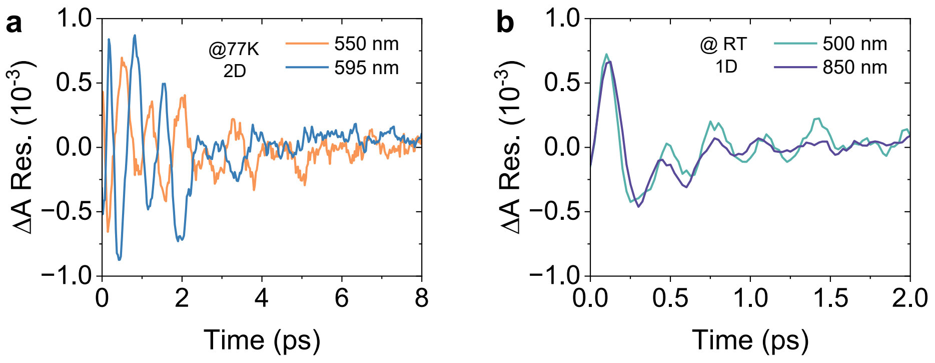

Supplementary Fig. 37 Excited state and ground state oscillatory signals. a The kinetics of GSB and ESA signals in the 2D system showing a phase shift of π. **b** The kinetics of ESA signals in the 1D system show no phase shift.

Supplementary Table 7 Assignment of the vibrational spectrum in the 1D system.

Mode	Exp. /cm^{-1}	Calc. /cm^{-1}	Mode assignment
M1	15	23	I-Sn-I rocking
M2	32	34	I-Sn-I twisting & rocking
M3	52	51	I-Sn-I twisting
M4	77	77	I-Sn-I twisting & asymmetric stretching
M5	106	104	I-Sn-I wagging & asymmetric stretching
M6	139	136	I-Sn-I scissoring

Supplementary References

1. Spanopoulos, I. *et al.* Water-stable 1d hybrid tin(II) iodide emits broad light with 36% photoluminescence quantum efficiency. *J. Am. Chem. Soc.* **142,** 9028-9038 (2020).
2. He, Y. *et al.* Unveiling mechanism of temperature-dependent self-trapped exciton emission in 1D hybrid organic–inorganic tin halide for advanced thermography. *Adv. Opt. Mater.* **13,** 2402061 (2024).
3. Pope, M. & Swenberg, C. E. *Electronic processes in organic crystals and polymers*. (Oxford University Press, 1999).
4. Griffiths, D. J. & Schroeter, D. F. *Introduction to quantum mechanics*. (Cambridge University Press, 2018).
5. Zwiebach, B. *Mastering quantum mechanics: Essentials, theory, and applications*. (MIT Press, 2022).
6. Loudon, R. *The quantum theory of light*. (OUP Oxford, 2000).
7. Huang, K. & Rhys, A. Theory of light absorption and non-radiative transitions in f-centres. *Proc. R. Soc. Lond. A Math. Phys. Sci.* **204,** 406-423 (1950).
8. Landau, L. D. & Lifshitz, E. M. *Quantum mechanics: Non-relativistic theory*, vol. 3. (Elsevier, 2013).
9. Köhler, A. & Bässler, H. *Electronic processes in organic semiconductors: An introduction*. (John Wiley & Sons, 2015).
10. Baranowski, M. *et al.* Static and dynamic disorder in triple-cation hybrid perovskites. *J. Phys. Chem. C* **122,** 17473-17480 (2018).
11. Dar, M. I. *et al.* Origin of unusual bandgap shift and dual emission in organic-inorganic lead halide perovskites. *Sci. Adv.* **2,** e1601156 (2016).
12. Kahmann, S., Shao, S. & Loi, M. A. Cooling, scattering, and recombination—the role of the material quality for the physics of tin halide perovskites. *Adv. Funct. Mater.* **29,** 1902963 (2019).
13. Lee, J., Koteles, E. S. & Vassell, M. O. Luminescence linewidths of excitons in GaAs quantum wells below 150 K. *Phys. Rev. B* **33,** 5512-5516 (1986).
14. Wright, A. D. *et al.* Electron–phonon coupling in hybrid lead halide perovskites. *Nat. Commun.* **7,** 11755 (2016).
15. Leroux, M. *et al.* Temperature quenching of photoluminescence intensities in undoped and doped GaN. *J. Appl. Phys.* **86,** 3721-3728 (1999).
16. Hulett, J. Deviations from the Arrhenius equation. *Chem. Soc. Rev.* **18,** 227-242 (1964).

17. Zhang, T. *et al.* Regulation of the luminescence mechanism of two-dimensional tin halide perovskites. *Nat. Commun.* **13,** 60 (2022).
18. Yuan, F. *et al.* Color-pure red light-emitting diodes based on two-dimensional lead-free perovskites. *Sci. Adv.* **6,** eabb0253 (2020).
19. Pitaro, M., Tekelenburg, E. K., Shao, S. & Loi, M. A. Tin halide perovskites: From fundamental properties to solar cells. *Adv. Mater.* **34,** 2105844 (2022).
20. Blancon, J. C. *et al.* Scaling law for excitons in 2D perovskite quantum wells. *Nat. Commun.* **9,** 2254 (2018).
21. Narra, S., Lin, C.-Y., Seetharaman, A., Jokar, E. & Diau, E. W.-G. Femtosecond exciton and carrier relaxation dynamics of two-dimensional (2D) and quasi-2D tin perovskites. *J. Phys. Chem. Lett.* **12,** 12292-12299 (2021).
22. Dyksik, M. *et al.* Steric engineering of exciton fine structure in 2D perovskites. *Adv. Energy Mater.* **15,** 2404769 (2025).
23. Lanzetta, L., Marin-Beloqui, J. M., Sanchez-Molina, I., Ding, D. & Haque, S. A. Two-dimensional organic tin halide perovskites with tunable visible emission and their use in light-emitting devices. *ACS Energy Lett.* **2,** 1662-1668 (2017).
24. Lin, R. *et al.* Dual self-trapped exciton emission with ultrahigh photoluminescence quantum yield in $CsCu_2I_3$ and $Cs_3Cu_2I_5$ perovskite single crystals. *J. Phys. Chem. C* **124,** 20469-20476 (2020).
25. Jun, T. *et al.* Lead-free highly efficient blue-emitting $Cs_3Cu_2I_5$ with 0D electronic structure. *Adv. Mater.* **30,** 1804547 (2018).
26. Luo, J. *et al.* Efficient and stable emission of warm-white light from lead-free halide double perovskites. *Nature* **563,** 541-545 (2018).
27. Nguyen, X. T. *et al.* Ultrafast charge carrier relaxation in inorganic halide perovskite single crystals probed by two-dimensional electronic spectroscopy. *J. Phys. Chem. Lett.* **10,** 5414-5421 (2019).
28. Blancon, J.-C. *et al.* Extremely efficient internal exciton dissociation through edge states in layered 2d perovskites. *Science* **355,** 1288-1292 (2017).
29. Mao, L. *et al.* Hybrid Dion–Jacobson 2D lead iodide perovskites. *J. Am. Chem. Soc.* **140,** 3775-3783 (2018).
30. Stoumpos, C. C. *et al.* Ruddlesden–Popper hybrid lead iodide perovskite 2D homologous semiconductors. *Chem. Mater.* **28,** 2852-2867 (2016).
31. Yang, Y. *et al.* Observation of a hot-phonon bottleneck in lead-iodide perovskites. *Nat. Photon.* **10,** 53-59 (2016).

32. Price, M. B. *et al.* Hot-carrier cooling and photoinduced refractive index changes in organic–inorganic lead halide perovskites. *Nat. Commun.* **6,** 8420 (2015).
33. Manser, J. S. & Kamat, P. V. Band filling with free charge carriers in organometal halide perovskites. *Nat. Photon.* **8,** 737-743 (2014).
34. Zheng, K. *et al.* High excitation intensity opens a new trapping channel in organic–inorganic hybrid perovskite nanoparticles. *ACS Energy Lett.* **1,** 1154-1161 (2016).
35. Stevens, T., Kuhl, J. & Merlin, R. Coherent phonon generation and the two stimulated Raman tensors. *Phys. Rev. B* **65,** 144304 (2002).
36. Bragas, A. V. *et al.* Ultrafast optical generation of coherent phonons in $CdTe_{1-x}Se_x$ quantum dots. *Phys. Rev. B* **69,** 205306 (2004).
37. Fu, J. *et al.* Electronic states modulation by coherent optical phonons in 2D halide perovskites. *Adv. Mater.* **33,** 2006233 (2021).
38. Ramesh, S. *et al.* Coherent phonons, localization, and slow polaron formation in lead-free gold perovskite. *Adv. Opt. Mater.* **13,** 2402882 (2025).
39. Coropceanu, V. *et al.* Charge transport in organic semiconductors. *Chem. Rev.* **107,** 926-952 (2007).
